# Structural Drift: The Population Dynamics of Sequential Learning

**DOI:** 10.1371/journal.pcbi.1002510

**Published:** 2012-06-07

**Authors:** James P. Crutchfield, Sean Whalen

**Affiliations:** 1 Complexity Sciences Center, Physics Department, University of California Davis, Davis, California, United States of America; 2 Computer Science Department, Columbia University, New York, New York, United States of America; University of Washington, United States of America

## Abstract

We introduce a theory of sequential causal inference in which learners in a chain estimate a structural model from their upstream “teacher” and then pass samples from the model to their downstream “student”. It extends the population dynamics of genetic drift, recasting Kimura's selectively neutral theory as a special case of a generalized drift process using structured populations with memory. We examine the diffusion and fixation properties of several drift processes and propose applications to learning, inference, and evolution. We also demonstrate how the organization of drift process space controls fidelity, facilitates innovations, and leads to information loss in sequential learning with and without memory.

## Author Summary

Human knowledge is often transmitted orally within a group via a sequence of communications between individuals. The children's game of *Telephone* is a familiar, simplified version. A phrase is uttered, understood, and then transmitted to another. Genetic information is communicated in an analogous sequential communication chain via replication. We show that the evolutionary dynamics of both problems is a form of genetic drift which accounts for memory in the communication chain. Using this, one can predict the mechanisms that lead to variations in fidelity and to structural innovation.

## Introduction

“Send Three- and Four-Pence, We're Going to a Dance”

This phrase was heard, it is claimed, over the radio during WWI instead of the transmitted tactical phrase “Send reinforcements we're going to advance” [1]. As illustrative as it is apocryphal, this garbled yet comprehensible transmission sets the tone for our investigations here. Namely, what happens to knowledge when it is communicated sequentially along a chain, from one individual to the next? What fidelity can one expect? How is information lost? How do innovations occur?

To answer these questions we introduce a theory of sequential causal inference in which learners in a communication chain estimate a structural model from their upstream “teacher” and then, using that model, pass along samples to their downstream “student”. This reminds one of the familiar children's game *Telephone*. By way of quickly motivating our sequential learning problem, let's briefly recall how the game works.

To begin, one player invents a phrase and whispers it to another player. This player, believing they have understood the phrase, then repeats it to a third and so on until the last player is reached. The last player announces the phrase, winning the game if it matches the original. Typically it does not, and that's the fun. Amusement and interest in the game derive directly from how the initial phrase evolves in odd and surprising ways. The further down the chain, the higher the chance that errors will make recovery impossible and the less likely the original phrase will survive.

The game is often used in education to teach the lesson that human communication is fraught with error. The final phrase, though, is not merely accreted error but the product of a series of attempts to parse, make sense, and intelligibly communicate the phrase. The phrase's evolution is a trade off between comprehensibility and accumulated distortion, as well as the source of the game's entertainment. We employ a much more tractable setting to make analytical progress on sequential learning, based on *computational mechanics* [2]–[4], intentionally selecting a simpler language system and learning paradigm than likely operates with children.

Specifically, we develop our theory of sequential learning as an extension of the evolutionary population dynamics of genetic drift, recasting Kimura's selectively neutral theory [5] as a special case of a generalized drift process of structured populations with memory. This is a substantial departure from the unordered populations used in evolutionary biology. Notably, this requires a new and more general information-theoretic notion of fixation. We examine the diffusion and fixation properties of several drift processes, demonstrating that the space of drift processes is highly organized. This organization controls fidelity, facilitates innovations, and leads to information loss in sequential learning and evolutionary processes with and without memory. We close by describing applications to learning, inference, and evolution, commenting on related efforts.

To get started, we briefly review genetic drift and fixation. This will seem like a distraction, but it is a necessary one since available mathematical results are key. Then we introduce in detail our structured variants of these concepts—defining the *generalized drift process* and formulating a generalized definition of fixation appropriate to it. With the background laid out, we begin to examine the complexity of structural drift behavior. We demonstrate that it is a diffusion process within a space that decomposes into a connected network of structured subspaces. Building on this decomposition, we explain how and when processes jump between these subspaces—innovating new structural information or forgetting it—thereby controlling the long-time fidelity of the communication chain. We then close by outlining future research and listing several potential applications for structural drift, drawing out consequences for evolutionary processes that learn.

Those familiar with neutral evolution theory are urged to skip to Section Sequential Learning, after skimming the next sections to pick up our notation and extensions.

### From Genetic to Structural Drift

Genetic drift refers to the change over time in genotype frequencies in a population due to random sampling. It is a central and well studied phenomenon in population dynamics, genetics, and evolution. A population of genotypes evolves randomly due to drift, but typically changes are neither manifested as new phenotypes nor detected by selection—they are *selectively neutral*. Drift plays an important role in the spontaneous emergence of mutational robustness [6], [7], modern techniques for calibrating molecular evolutionary clocks [8], and nonadaptive (neutral) evolution [9], [10], to mention only a few examples.

Selectively neutral drift is typically modeled as a stochastic process: A random walk that tracks finite populations of individuals in terms of their possessing (or not) a variant of a gene. In the simplest models, the random walk occurs in a space that is a function of genotypes in the population. For example, a drift process can be considered to be a random walk of the *fraction* of individuals with a given variant. In the simplest cases there, the model reduces to the dynamics of repeated binomial sampling of a biased coin, in which the empirical estimate of bias becomes the bias in the next round of sampling. In the sense we will use the term, the sampling process is *memoryless*. The biased coin, as the population being sampled, has no memory: The past is independent of the future. The current state of the drift process is simply the bias, a number between zero and one that summarizes the state of the population.

The theory of genetic drift predicts a number of measurable properties. For example, one can calculate the expected time until all or no members of a population possess a particular gene variant. These final states are referred to as *fixation* and *deletion*, respectively. Variation due to sampling vanishes once these states are reached and, for all practical purposes, drift stops. From then on, the population is homogeneous; further sampling can introduce no genotypic variation. These states are fixed points—in fact, absorbing states—of the drift stochastic process.

The analytical predictions for the time to fixation and time to deletion were developed by Kimura and Ohta [5], [11] in the 1960s and are based on the memoryless models and simplifying assumptions introduced by Wright [12] and Fisher [13] in the early 1930s. The theory has advanced substantially since then to handle more realistic models and to predict additional effects due to selection and mutation. These range from multi-allele drift models and 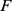-statistics [14] to pseudohitchhiking models of “genetic draft” [15].

The following explores what happens when we relax the memoryless restriction. The original random walk model of genetic drift forces the statistical structure at each sampling step to be an independent, identically distributed (IID) stochastic process. This precludes any memory in the sampling. Here, we extend the IID theory to use time-varying probabilistic state machines to describe memoryful population sampling.

In the larger setting of sequential learning, we will show that memoryful sequential sampling exhibits structurally complex, drift-like behavior. We call the resulting phenomenon *structural drift*. Our extension presents a number of new questions regarding the organization of the space of drift processes and how they balance structure and randomness. To examine these questions, we require a more precise description of the original drift theory.

### Genetic Drift

We begin with the definition of an *allele*, which is one of several alternate forms of a gene. The textbook example is given by Mendel's early experiments on heredity [16], in which he observed that the flowers of a pea plant were colored either white or violet, this being determined by the combination of alleles inherited from its parents. A new, *mutant* allele is introduced into a population by the mutation of a *wild-type* allele. A mutant allele can be passed on to an individual's offspring who, in turn, may pass it on to their offspring. Each inheritance occurs with some probability.

*Genetic drift*, then, is the change of allele frequencies in a population over time. It is the process by which the number of individuals with an allele varies generation after generation. The Fisher-Wright theory [12], [13] models drift as a stochastic evolutionary process with neither selection nor mutation. It assumes random mating between individuals and that the population is held at a finite, constant size. Moreover, successive populations do not overlap in time.

Under these assumptions the Fisher-Wright theory reduces drift to a binomial or multinomial sampling process—a more complicated version of familiar random walks such as Gambler's Ruin or Prisoner's Escape [17]. Offspring receive either the wild-type allele 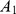 or the mutant allele 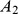 of a particular gene 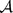 from a random parent in the previous generation with replacement. A population of 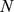 diploid individuals will have 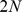 total copies of these alleles. (Though we first use diploid populations (two alleles per individual and thus a sample length of 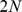) for direct comparison to previous work, we later transition to haploid (single allele per individual) populations for notational simplicity.) Given 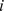 initial copies of 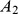 in the population, an individual has either 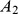 with probability 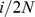 or 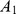 with probability 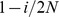. The probability that 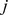 copies of 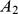 exist in the offspring's generation given 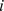 copies in the parent's generation is:
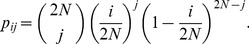
(1)This specifies the transition dynamic of the drift stochastic process over the discrete state space
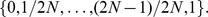


This model of genetic drift is a discrete-time random walk, driven by samples of a biased coin, over the space of biases. The population is a set of coin flips, where the probability of HEADS or TAILS is determined by the coin's current bias. After each generation of flips, the coin's bias is updated to reflect the number of HEADS or TAILS realized in the new generation. The walk's absorbing states—all HEADS or all TAILS—capture the notion of fixation and deletion.

### Genetic Fixation

*Fixation* occurs with respect to an allele when all individuals in the population carry that specific allele and none of its variants. Restated, a mutant allele 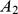 reaches fixation when all 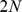 alleles in the population are copies of 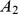 and, consequently, 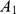 has been *deleted* from the population. This halts the random fluctuations in the frequency of 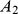, assuming 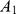 is not reintroduced.

Let 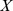 be a binomially distributed random variable with bias probability 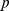 that represents the fraction of copies of 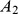 in the population. The expected number of copies of 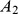 is 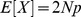. That is, the expected number of copies of 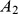 remains constant over time and depends only on its initial probability 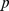 and the total number (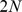) of alleles in the population. However, 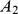 eventually reaches fixation or deletion due to the change in allele frequency introduced by random sampling and the presence of absorbing states. Prior to fixation, the mean and variance of the change in allele frequency 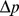 are:
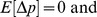
(2)

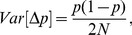
(3)respectively.

On average there is no change in frequency. However, sampling variance causes the process to drift towards the absorbing states at 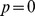 and 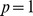. The drift rate is determined by the current generation's allele frequency and the total number of alleles. For the neutrally selective case, the average number of generations until fixation (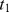) or deletion (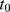) is given by Kimura and Ohta [5]:
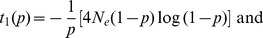
(4)

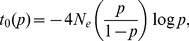
(5)where 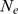 denotes effective population size. For simplicity we take 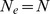, meaning all individuals in the population are candidates for reproduction. As 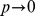, the boundary condition is given by:
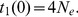
(6)That is, excluding cases of deletion, an initially rare mutant allele spreads to the entire population in 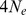 generations.

One important consequence of the theory is that when fixation (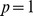) or deletion (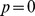) are reached, variation in the population vanishes: 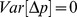. With no variation there is a homogeneous population, and sampling from this population produces the same homogeneous population. In other words, this establishes fixation and deletion as absorbing states of the stochastic sampling process. Once there, drift stops.

**Figure 1:**
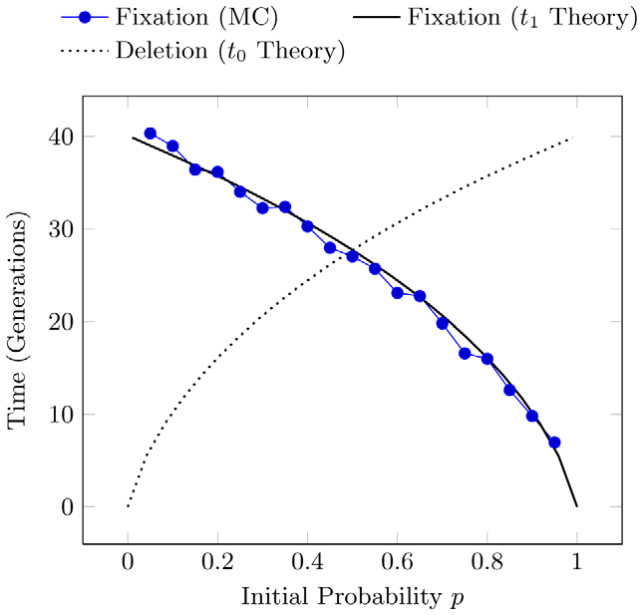
Time to fixation for a population of 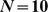 individuals (sample size 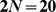) plotted as a function of initial allele probability 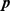 under the Monte Carlo (MC) sampling regime and as given by theoretical prediction (solid line) of Eq. (4). Time to deletion is also shown (dashed line), Eq. (5).

Figure 1 illustrates this, showing both the simulated and theoretically predicted number of generations until fixation occurs for 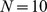, as well as the predicted time to deletion for reference. Each simulation was performed for a different initial value of 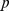 and averaged over 400 realizations. Using the same methodology as Kimura and Ohta [5], we include only those realizations whose mutant allele reaches fixation.

Populations are produced by repeated binomial sampling of 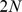 uniform random numbers between 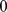 and 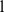. An initial probability 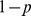 is assigned to allele 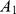 and probability 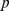 to allele 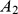. The count 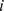 of 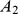 in the initial population is incremented for each random number less than 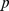. This represents an individual acquiring the allele 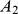 instead of 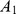. The maximum likelihood estimate of allele frequency in the initial sample is simply the number of 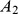 alleles over the sample length: 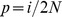. This estimate of 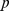 is then used to generate a new population of offspring, after which we re-estimate the value of 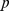. These steps are repeated each generation until fixation at 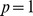 or deletion at 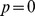 occurs. This is the *Monte Carlo* (MC) sampling method.

Kimura's theory and simulations predict the time to fixation or deletion of a mutant allele in a finite population by the process of genetic drift. The Fisher-Wright model and Kimura's theory assume a memoryless population in which each offspring inherits allele 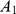 or 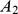 via an IID binomial sampling process. We now generalize this to memoryful stochastic processes, giving a new definition of fixation and exploring examples of structural drift behavior.

## Methods

### Sequential Learning

How can genetic drift be a memoryful stochastic process? Consider a population of 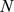 haploid organisms. Each generation consists of 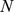 alleles and so is represented by a string of 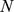 symbols, e.g. 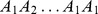, where each symbol corresponds to an individual with a particular allele. In the original drift models, a generation of offspring is produced by a memoryless binomial sampling process, selecting an offspring's allele from a parent with replacement. In contrast, the structural drift model produces a generation of individuals in which the sample order is tracked. The population is now a string of alleles, giving the potential for memory and structure in sampling—spatial, temporal, or other interdependencies between individuals within a sample.

At first, this appears as a major difference from the usual setting employed in population biology, where populations are treated as unordered collections of individuals and sampling is modeled as an independent, identically distributed stochastic process. That said, the structure we have in mind has several biological interpretations, such as inbreeding and subdivision [18] or the life histories of heterogeneous populations [19]. We later return to these alternative interpretations when considering applications.

The model class we select to describe memoryful sampling is the 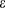-machine : the unique, minimal, and optimal representation of a stochastic process [4]. As we will show, these properties give an important advantage when analyzing structural drift, since they allow one to monitor the amount of structure innovated or lost during drift. We next give a brief overview of 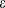-machines and refer the reader to the previous reference for details.

The 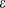-machine representations of the finite-memory discrete-valued stochastic processes we consider here form a class of (deterministic) probabilistic finite-state machine or unifilar hidden Markov model. An 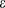-machine consists of a set of *causal states* 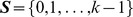 and a set of per-symbol transition matrices:
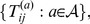
(7)where 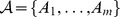 is the set of alleles and where the transition probability 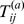 gives the probability of transitioning from causal state 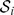 to causal state 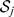 and emitting allele 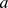. The causal state probability 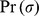, 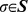, is determined as the left eigenvector of the state-to-state transition matrix 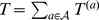.

Maintaining our connection to (haploid) population dynamics, we think of an 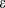-machine as a generator of populations or length-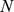 strings: 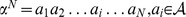. As a model of a sampling process, an 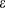-machine gives the most compact representation of the distribution of strings produced by sampling.

**Figure 2:**
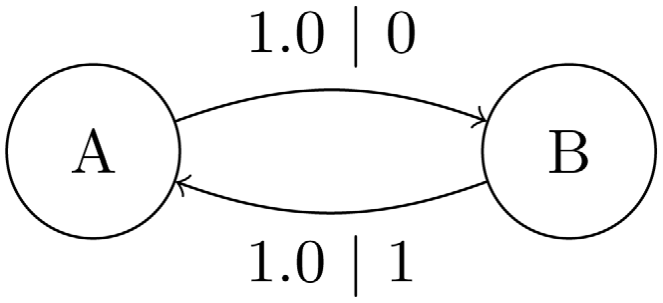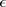-Machine for the Alternating Process consisting of two causal states 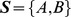 and two transitions. State 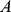 emits allele 0 with probability one and transitions to state 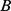, while 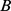 emits allele 1 with probability one and transitions to 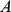.

Consider a simple binary process that alternately generates 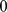
s and 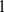
s called the *Alternating Process* shown in Figure 2. Its 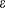-machine generates either the string 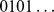 or 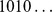 depending on the start state. The per-symbol transition matrices are:
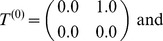
(8)

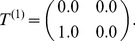
(9)


**Figure 3:**
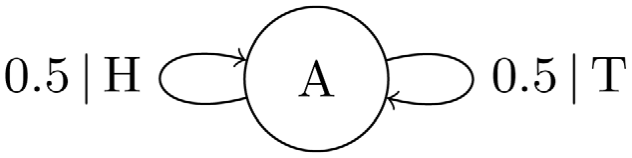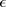-Machine for the Fair Coin Process consisting of a single causal state 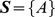 and a self-transition for both HEADS and TAILS. Each transition is labeled 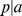 to indicate the probability 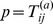 of taking that transition and emitting allele 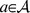. We refer to the Biased Coin Process when 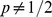.

**Figure 4:**
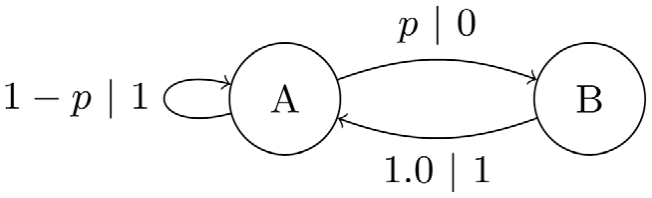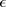-Machine for the Golden Mean Process consisting of two causal states 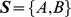 that generates a population with no consecutive 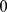
s. In state 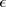 the probabilities of generating a 0 or 1 are 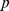 and 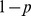, respectively.

Enforcing the alternating period-2 pattern requires two states, 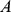 and 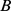, as well as two positive probability transitions 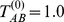 and 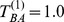. Branching transitions are required for a process to structurally drift; the Alternating Process has none. Two simple 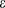-machines with branching structure are the smaller Fair Coin Process (Figure 3) and more complex Golden Mean Process (Figure 4). Both are discussed in detail later.

Beyond using 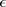-machines as generators of stochastic processes, as just described, several alternative *reconstruction* algorithms exist to infer 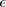-machines from data samples—tree-merging [2], state-splitting [20], and spectral [21]. These algorithms share a general approach: First, estimate the distribution of subsequences. (If given data as a single string, for example, slide a window of length 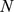 over the string and count subsequences of lengths 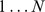.) Second, compute the distinct probability distributions of future subsequences conditioned on past subsequences (histories). Third, partition histories into equivalence classes (causal states) that give the same conditional future distributions. And, finally, calculate the transition dynamic between states. Properly reconstructed, the causal states form a minimal sufficient statistic for prediction in the sense of Kullback [22]. Here, we circumvent these methods' complications. Section Structural Innovation and Loss introduces an alternative that avoids them and is, at the same time, more computationally efficient.

**Figure 5:**
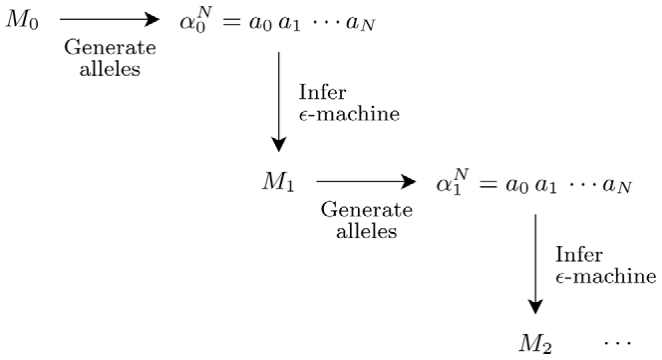
Sequential inference with a chain of 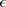-machines. An initial population generator 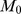 produces a length-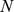 string 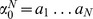 from which a new model 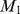 is inferred. These steps are repeated using 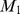 as the population generator and so on, until a terminating condition is met.

We are now ready to describe *sequential learning*, depicted in Figure 5. We begin by selecting the 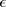-machine 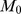 as an initial population generator. Following a path through 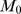, guided by its transition probabilities, produces a length-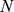 string 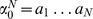 that represents the first population of 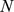 individuals possessing alleles 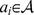. We then infer an 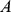-machine 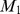 from the population 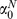. 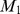 is then used to produce a new population 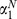, from which a new 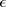-machine 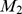 is estimated. This new population has the same allele distribution as the previous, plus some amount of variance. The cycle of inference and re-inference is repeated while allele frequencies drift each generation until fixation or deletion is reached. At that point, the populations (and so 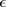-machines ) cannot vary further. The net result is a stochastically varying time series of 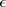-machines (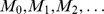) that terminates when the populations 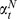 stop changing.

Thus, at each step a new representation or model is estimated from the previous step's sample. The inference step highlights that this is learning: a model of the generator is estimated from the given finite data. The repetition of this step creates a sequential communication chain. Sequential learning is thus closely related to genetic drift except that sample order is tracked, and this order is used in estimating the next generator.

The procedure is analogous to flipping a biased coin a number of times, estimating the bias from the results, and re-flipping the newly biased coin. Eventually, the coin will be completely biased towards Heads or Tails. In our drift model the coin is replaced by an 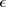-machine, which removes the IID model constraint and allows for the sampling process to take on structure and memory. Not only do the transition probabilities 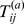 change, but *the structure of the generator itself*—the number of states and the presence or absence of transitions—drifts over time to capture the statistics of the sample using as little information as possible. This is an essential and distinctive aspect of structural drift.

Before we can explore this dynamic, we first need to examine how an 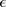-machine reaches fixation or deletion.

### Structural Stasis

Recall the Alternating Process from Figure 1, producing the strings 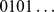 and 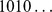 depending on the start state. Regardless of the initial state, the original 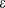-machine is re-inferred from any sufficiently long string it produces. In the context of sequential learning, this means the population at each generation is the same.

However, if we consider allele 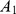 to be represented by symbol 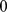 and 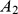 by symbol 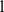, neither allele reaches fixation or deletion according to current definitions. Nonetheless, the Alternating Process prevents any variance between generations and so, despite the population not being all 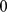 s or all 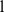 s, the population does reach an equilibrium: half 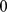 s and half 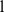 s. For these reasons, one cannot use the original population-dynamics definitions of fixation and deletion.

This leads us to introduce *structural stasis* to combine the notions of fixation, deletion, and the inability to vary caused by periodicity. Said more directly, structural stasis corresponds to a process becoming nonstochastic, since it ceases to introduce variance between generations and so prevents further drift. However, we need a method to detect the occurrence of structural stasis in a drift process.

A state machine representing a periodic sampling process enforces the constraint of periodicity via its internal memory. One measure of this memory is the *population diversity* 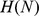 [23]:
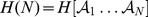
(10)

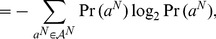
(11)where the units are [bits]. (For background on information theory as used here, the reader is referred to Ref. [24].) The population diversity of the Alternating Process is 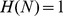 bit at any size 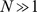. This single bit of information corresponds to the machine's current phase or state. Generally, though, the value diverges—
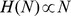
—for arbitrary sampling processes, and so population diversity is not suitable as a general test for stasis.

Instead, the condition for stasis can be given as the vanishing of the *growth rate* of population diversity:
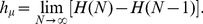
(12)Equivalently, we can test the per-allele entropy of the sampling process. We call this *allelic entropy*:
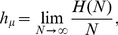
(13)where the units are [bits per allele]. Allelic entropy gives the average information per allele in bits, and structural stasis occurs when 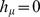. While closer to a general test for stasis, this quantity is difficult to estimate from population samples since it relies on an asymptotic estimate of the population diversity. However, the allelic entropy can be calculated in closed-form from the 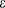-machine representation of the sampling process:
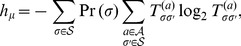
(14)For example, the Alternating Process has 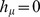, the Fair Coin Process 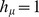, and the Golden Mean Process 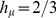; all in units of bits per symbol. When 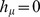, the sampling process has become periodic and lost all randomness generated via its branching transitions. In this way, we replace the vanishing variance (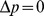) of a single bias parameter in the Kimura drift setting with a general measure of the sampling process's stochasticity. This new criterion subsumes the notions of fixation and deletion as well as periodicity. An 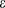-machine has zero allelic entropy if any of these conditions occur. More formally, we have the following statement.

**Definition** Structural stasis *occurs when the sampling process's allelic entropy vanishes:* 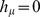 *.*

**Proposition** *Structural stasis is a fixed point of finite-memory structural drift.*

**Proof** *Finite-memory means that the* 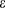 *-machine representing the population sampling process has a finite number of states. Given this, if* 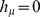 *, then the* 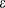 *-machine has no branching in its recurrent states:* 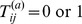 *, where* 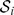 *and* 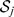 *are asymptotically recurrent states. This results in no variation in the inferred* 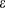 *-machine when sampling sufficiently large populations. Lack of variation, in turn, means the transition probabilities can no longer change and so the drift process stops. If allelic entropy vanishes at time* 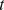 *and no mutations are allowed, then it is zero for all* 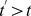 *. Thus, structural stasis is an absorbing state of the drift stochastic process.*

## Results

While more can be said analytically about structural drift, our present purpose is to introduce the main concepts. We will show that structural drift leads to interesting and nontrivial behavior. First, we calibrate the new class of drift processes against the original genetic drift theory.

### Memoryless Drift

The Biased Coin Process is represented by a single-state 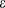-machine with a self loop for both Heads and Tails symbols; see Figure 3. It is an IID sampling process that generates populations with a binomial distribution of alleles. Unlike the Alternating Process, the coin's bias 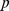 is free to drift during sequential inference. These properties make the Biased Coin Process an ideal candidate for exploring memoryless drift.

**Figure 6:**
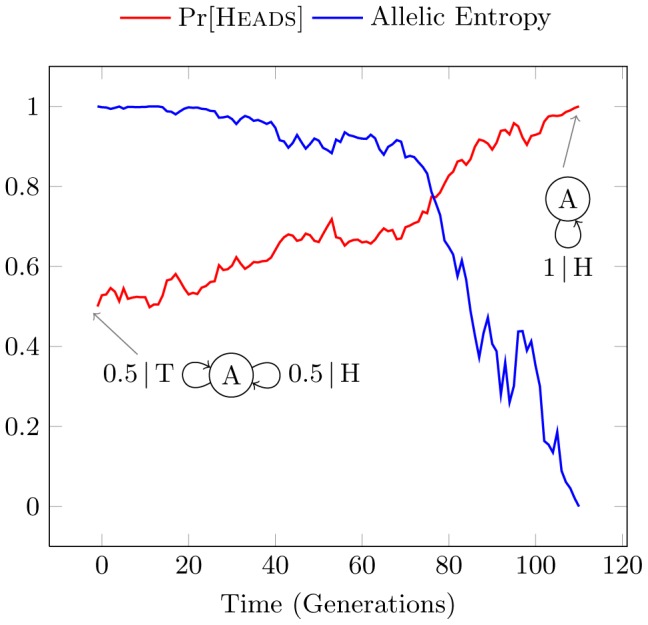
Drift of allelic entropy 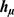 and Pr[Heads] for a single realization of the Biased Coin Process with sample size 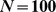. The drift of Pr[Heads] is annotated with its initial machine 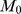 (left inset) and the machine at stasis 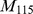 (right inset).

Figure 6 shows structural drift, using two different measures, for a single realization of the Biased Coin Process with initial 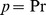 [Heads] = Pr [Tails] = 0.5. Structural stasis (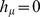) is reached after 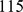 generations. The initial Fair Coin 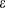-machine occurs at the left of Figure 6 and the final, completely biased 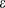-machine occurs at the right.

Note that the drift of allelic entropy 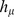 and 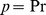 [Tails] are inversely related, with allelic entropy converging quickly to zero as stasis is approached. This reflects the rapid drop in population diversity. After stasis occurs, all randomness has been eliminated from the transitions at state 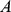, resulting in a single transition that always produces TAILS. Anticipating later discussion, we note that during this run only Biased Coin Processes were observed.

**Figure 7:**
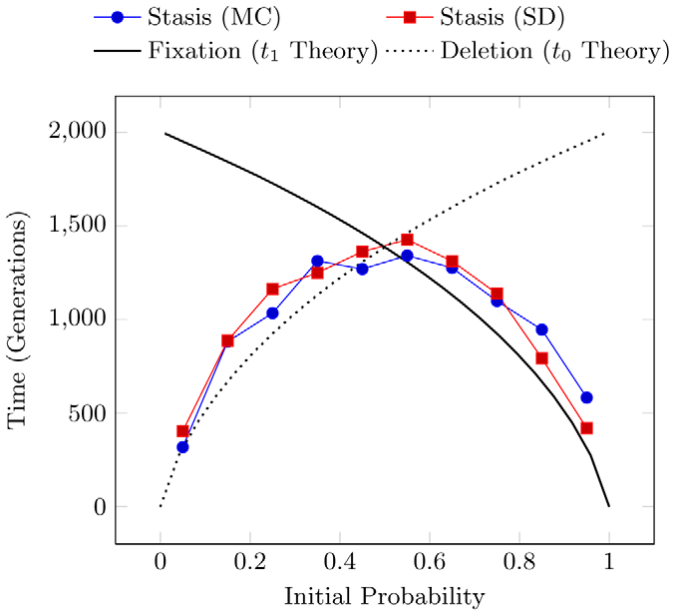
Time to stasis as a function of initial Pr[Heads] for structural drift (SD) of the Biased Coin Process versus Monte Carlo (MC) simulation of Kimura's model. Kimura's predicted times to fixation and deletion are shown for reference. Each estimated time is averaged over 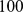 realizations with sample size 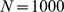.

The time to stasis of the Biased Coin Process as a function of initial 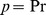 [Heads] was shown in Figure 7. Also shown there was the previous Monte Carlo Kimura drift simulation modified to terminate when either fixation or deletion occurs. This experiment illustrates the definition of structural stasis and allows direct comparison of structural drift with genetic drift in the memoryless case.

Not surprisingly, we can interpret genetic drift as a special case of the structural drift process for the Biased Coin. Both simulations follow Kimura's theoretically predicted curves, combining the lower half of the deletion curve with the upper half of the fixation curve to reflect the initial probability's proximity to the absorbing states. A high or low initial bias leads to a shorter time to stasis as the absorbing states are closer to the initial state. Similarly, a Fair Coin is the furthest from absorption and thus takes the longest average time to reach stasis.

### Structural Drift

The Biased Coin Process represents an IID sampling process with no memory of previous flips, reaching stasis when Pr[Heads] = 1.0 or 0.0 and, correspondingly, when 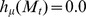. We now introduce memory by starting drift with 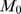 as the *Golden Mean Process*, which produces binary populations with no consecutive 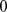
s. Its 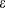-machine was shown in Figure 4. Note that one can initialize drift using any stochastic process; for example, see the 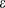-machine library of Ref. [25].

Like the Alternating Process, the Golden Mean Process has two causal states. However, the transitions from state 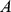 have nonzero entropy, allowing their probabilities to drift as new 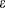-machines are inferred from generation to generation. If the 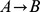 transition probability 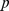 (Figure 4) becomes zero the transition is removed, and the Golden Mean Process reaches stasis by transforming into the Fixed Coin Process (top right, Figure 6). Instead, if the same transition drifts towards probability 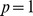, the 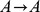 transition is removed. In this case, the Golden Mean Process reaches stasis by transforming into the Alternating Process (Figure 2).

**Figure 8:**
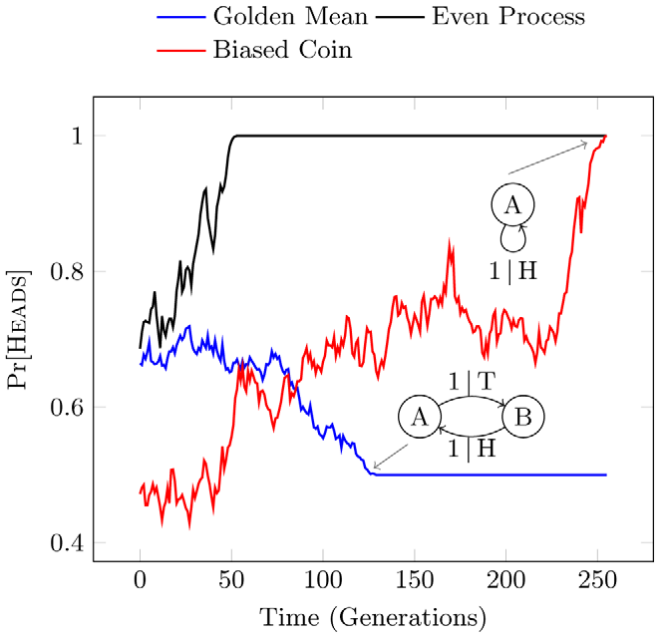
Drift of Pr[Heads] for a single realization of the Biased Coin, Golden Mean, and Even Processes, plotted as a function of generation. The Even and Biased Coin Processes become the Fixed Coin Process at stasis, while the Golden Mean Process becomes the Alternating Process. Note that the definition of structural stasis recognizes the lack of variance in the Alternating Process subspace even though the allele probability is neither 0 nor 1.

To compare structural drift behaviors, consider also the Even Process. Similar in form to the Golden Mean Process, the Even Process produces populations in which blocks of consecutive 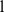
s must be even in length when bounded by 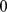
s [24]. Figure 8 compares the drift of Pr[Heads] for a single realization of the Biased Coin, Golden Mean, and Even Processes. One observes that the Even and Biased Coin Processes reach stasis as the Fixed Coin Process, while the Golden Mean Process reaches stasis as the Alternating Process. For different realizations, the Even and Golden Mean Processes might instead reach different stasis points.

**Figure 9:**
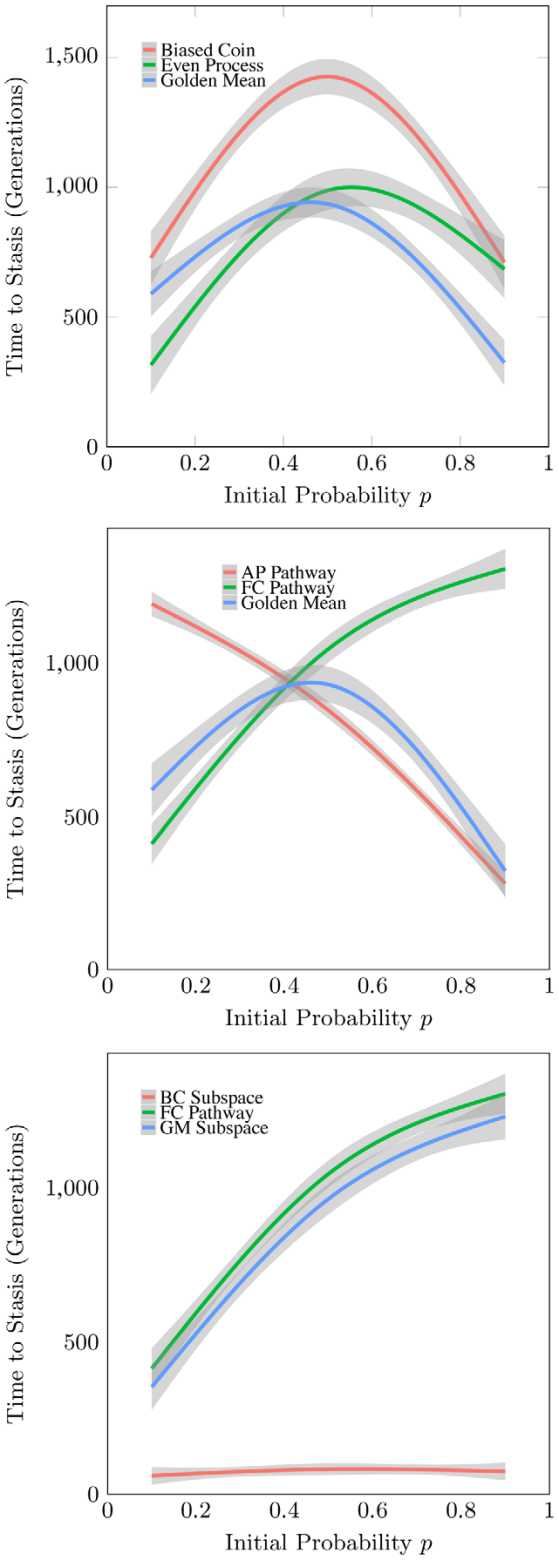
*Top:* Time to stasis of the Golden Mean, Even, and Biased Coin Processes. *Middle*: Stasis time of the Golden Mean Process as the weighted sum of stasis times for the Fixed Coin (FC) and Alternating Process (AP) pathways. *Bottom*: Stasis time of the FC pathway as the weighted sum of Golden Mean (GM) and Biased Coin (BC) subspace diffusion times.

It should be noted that the memoryful Golden Mean and Even Processes reach stasis markedly faster than the memoryless Biased Coin. While Figure 8 shows only a single realization of each sampling process type, the top panel of Figure 9 shows the large disparity in stasis times holds across all settings of each process's initial bias. This is one of our first general observations about memoryful processes: The structure of memoryful processes substantially impacts the average time to stasis by increasing variance between generations. In the cases shown, time to stasis is greatly shortened.

### Isostructural Subspaces

To illustrate the richness of structural drift and to understand how it affects average time to stasis, we examine the complexity-entropy (CE) diagram [26] of the 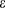-machines produced over several realizations of an arbitrary sampling process. The CE diagram displays how the allelic entropy 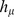 of an 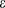-machine varies with the allelic complexity 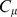 of its causal states:
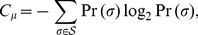
(15)where the units are [bits]. The allelic complexity is the Shannon entropy over an 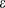-machine 's stationary state distribution 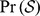. It measures the memory needed to maintain the internal state while producing stochastic outputs. 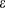-Machine minimality guarantees that 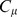 is the smallest amount of memory required to do so. Since there is a one-to-one correspondence between processes and their 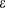-machines, a CE diagram is a projection of process space onto the two coordinates 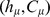. Used in tandem, these two properties differentiate many types of sampling process, capturing both their intrinsic memory (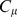) and the diversity (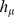) of populations they generate.

#### Subspace diffusion

**Figure 10:**
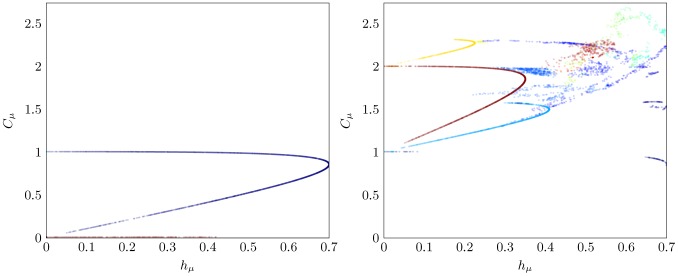
Complexity-entropy diagram for 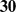 realizations of the Golden Mean Process with 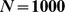, both without (left) and with (right) structural innovation. Alternating Process and Fixed Coin pathways are clearly visible in the left panel where the Golden Mean subspace exists on the upper curve and the Biased Coin subspace exists on the line 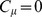. 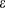-Machines within the same isostructural subspace have identical colors.

Two such CE diagrams are shown in Figure 10, illustrating different subspaces and stasis points reachable by the Golden Mean Process during structural drift. Consider the left panel first. An 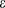-machine reaches stasis by transforming into either the Fixed Coin or the Alternating Process. To reach the former, the 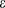-machine begins on the upper curve in the left panel and drifts until the 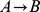 transition probability nears zero and the inference algorithm decides to merge states in the next generation. This forces the 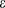-machine to jump to the Biased Coin subspace on the line 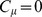 where it will most likely diffuse until the Fixed Coin stasis point at 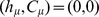 is reached. If instead the 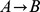 transition probability drifts towards zero, the Golden Mean stays on the upper curve until reaching the Alternating Process stasis point at 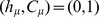. Thus, the two stasis points are differentiated not by 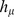 but by 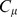, with the Alternating Process requiring 1 bit of memory to track its internal state and the Biased Coin Process requiring none.

What emerges from these diagrams is a broader view of how population structure drifts in process space. Roughly, the 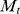 diffuse locally in the parameter space specified by the current, fixed architecture of states and transitions. During this, transition probability estimates vary stochastically due to sampling variance. Since 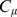 and 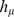 are continuous functions of the transition probabilities, this variance causes the 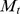 to fall on well defined curves or regions corresponding to a particular process subspace. (See Figures 4 and 5 in Ref. [26] and the theory for these curves and regions there.)

We refer to these curves as *isostructural curves* and the associated sets of 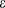-machines as *isostructural subspaces*. They are metastable subspaces of sampling processes that are quasi-invariant under the structural drift dynamic. When one or more 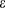-machine parameters diffuse sufficiently so that inference is forced to change topology by adding or removing states or transitions to reflect the statistics of the sample, this quasi-invariance is broken. We call such topological shifts *subspace jumps* to reflect the new region occupied by the resulting 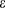-machine in process space, as visualized by the CE diagram. Movement between subspaces is often not bidirectional—innovations from a previous topology may be lost either temporarily (when the innovation can be restored by returning to the subspace) or permanently. For example, the Golden Mean subspace commonly jumps to the Biased Coin subspace but the opposite is highly improbable without mutation. (We consider the latter type of structural drift elsewhere.)

Before describing the diversity seen in the CE diagram of Figure 10's right panel, we first turn to analyze in some detail the time-to-stasis underlying the behavior illustrated in the left panel.

#### Subspace decomposition

A *pathway* is a set of subspaces passed through by any drift realization starting from some initial process and reaching a specific stasis point. The time to stasis of a drift process 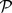 is the sum of time spent in the subspaces 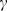 visited by its pathways to stasis 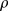, weighted by the probabilities that these pathways and subspaces will be reached. The time spent in a subspace 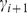 merely depends on the transition parameter(s) of the 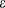-machine at the time of entry and is otherwise independent of the prior subspace 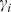. Thus, calculating the stasis time of a structured population can be broken down into independent subspace times when we know the values of the transition parameters at subspace jumps. These values can be derived both empirically and analytically, and we aim to develop the latter for general drift processes in future work.

More formally, the time to stasis 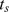 of a drift process 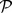 is simply the weighted sum of the stasis times for its connected pathways 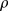:
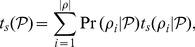
(16)Similarly, the stasis time of a particular pathway decomposes into the time spent diffusing in its connected subspaces 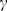:
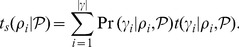
(17)To demonstrate, Figure 9 shows the stasis time of the Golden Mean Process (GMP) with initial bias 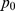 in more detail. Regression lines along with their 95% confidence intervals are displayed for simulations with initial biases 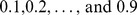. The middle panel shows the total time to stasis as the weighted sum of its Fixed Coin (FC) and Alternating Process (AP) pathways:
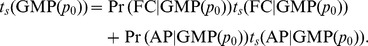
For low 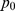, the transition from state 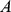 to state 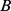 is unlikely, so 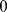 s are rare and the AP pathway is reached infrequently. Thus, the total stasis time is initially dominated by the FC pathway (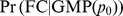 is high). As 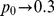 and above, the AP pathway is reached more frequently (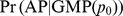 grows) and its stasis time begins to influence the total. The FC pathway is less likely as 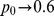 and the total time becomes dominated by the AP pathway (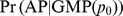 is high).

Since the AP pathway visits only one subspace, the bottom panel shows the stasis time of the FC pathway as the weighted sum of the Golden Mean (GM) and Biased Coin (BC) subspace times:
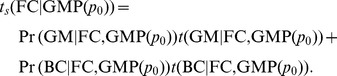
(18)This corresponds to time spent diffusing in the GM subspace *before* the subspace jump and time spent diffusing in the BC subspace *after* the subspace jump. Note that the times quoted are simply diffusion times within a subspace, since not every subspace in a pathway contains a stasis point.

These expressions emphasize the dependence of stasis time on the transition parameters at jump points as well as on the architecture of isostructural subspaces in drift process space. For example, if the GM jumps to the BC subspace at 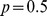, the stasis time will be large since the 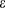-machine is maximally far from either stasis point. However, the inference algorithm will typically jump at very low values of 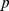 resulting in a small average stasis time for the BC subspace in the FC pathway. Due to this, calculating the stasis time for the GMP requires knowing the AP and FC pathways as well as the value of 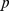 where the GM
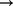
BC jump occurs.

#### Structural innovation and loss

Inference of 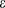-machines from finite populations is computationally expensive, particularly in our sequential setting with many realizations. The topology of the 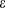-machine is inferred directly from the statistics of finite samples; both states and transitions are added and removed over time to capture innovation and loss of population structure. In the spirit of Kimura's *pseudo-sampling variable* (PSV) method [27], we introduce a PSV algorithm for efficient structural drift simulation and increased control of the trade-off between structural innovation and loss.

Instead of inferring and re-inferring an 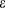-machine each generation, we explicitly define the conditions for topological changes to the 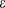-machine of the previous generation. To test for *structural innovation*, a random causal state from the current 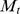 is cloned and random incoming transitions are routed instead to the cloned state. This creates a new model 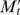 that describes the same process. Gaussian noise is then added to the cloned state's outgoing transitions to represent some change in population structure. The likelihood of the population 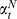 is calculated for both 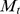 and 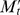 and the model with the maximum a posteriori (MAP) likelihood is retained:
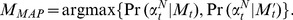
(19)If the original 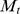 was retained, its transition parameters are updated by feeding the sample through the model to obtain edge counts which are then normalized to obtain probabilities. This produces a generator for the next generation's population in a way that allows for innovation. As well, it side-steps the computational cost of the inference algorithm.

To capture structural loss, we monitor near-zero transition probabilities where an 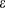-machine inference algorithm would merge states. When such a transition exists we test for structural simplification by considering all pairwise mergings of causal states and select the topology via the MAP likelihood. However, unlike above, we penalize likelihood using the Akaike Information Criterion (AIC) [28]:
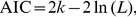
(20)and, in particular, the AIC corrected for finite sample sizes [29]:
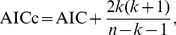
(21)where 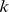 is the number of model parameters, 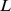 is the sample likelihood, and 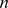 is the sample size. A penalized likelihood is necessary because a smaller 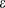-machine is more general and cannot fit the data as well. When penalized by model size, however, a smaller model with sufficient fit to the data may be selected over a larger, better fitting model. This method allows loss to occur while again avoiding the expense of the full 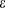-machine inference algorithm. Extensive comparisons with several versions of the latter show that the new PSV structural drift algorithm produces qualitatively the same behavior.

Having explained how the pseudo-drift algorithm introduces structural innovation and loss we can now describe the drift runs of Figure 10's right panel. In contrast to the left panel, structural innovation was enabled. The immediate result is that the drift process visits a much wider diversity of isostructural subspaces—sampling processes that are markedly more complex. 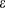-Machines with 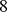 or more states are created, some of which are quite entropic and so produce high sampling variance. Stasis 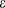-machines with periods 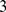, 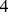, 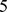, and 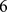 are seen, while only those with periods 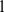 and 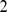 are seen in runs without innovation (left panel).

By way of closing this first discussion of structural drift, it should be emphasized that none of the preceding phenomena occur in the limit of infinite populations or infinite sample size. The variance due to finite sampling drives sequential learning, the diffusion through process space, and the jumps between isostructural subspaces.

## Discussion

### Applications and Extensions

Much of the previous discussion focused on structural drift as a kind of stochastic process, with examples and behaviors selected to emphasize the role of structure. Although there was a certain terminological bias toward neutral evolution theory since the latter provides an entree to analyzing how structural drift works, our presentation was intentionally general. Motivated by a variety of potential applications and extensions, we describe these now and close with several summary remarks on structural drift itself.

#### Emergent semantics and learning in communication chains

Let's return to draw parallels with the opening example of the game of *Telephone* or, more directly, to the sequential inference of temporal structure in an utterance passed along a serially coupled communication chain. There appears to be no shortage of related theories of language evolution. These range from the population dynamics of Ref. [30] and the ecological dynamics of Ref. [31] to the cataloging of error sources in human communication [32] and recent efforts to understand cultural evolution as reflecting learning biases [33], [34].

By way of contrast, structural drift captures the language-centric notion of dynamically changing semantics and demonstrates how behavior is driven by finite-sample fluctuations within a semantically organized subspace. The symbols and words in the generated strings have a semantics given by the structure of a subspace's 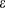-machine ; see Ref. [3]. A particularly simple example was identified quite early in the information-theoretic analysis of natural language: The Golden Mean 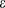-machine (Figure 4) describes the role of isolated space symbols in written English [35, Figure 1]. Notably, this structure is responsible for the Mandelbrot-Zipf power-law scaling of word frequencies [36], [37]. More generally, though, the semantic theory of 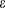-machines shows that causal states provide dynamic contexts for interpretation as individual symbols and words are recognized. Quantitatively, the allelic complexity 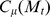 is the total amount of semantic content that can be generated by an 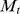 [3]. In this way, shifts in the architecture of the 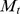 during drift correspond to semantic changes. That is, diffusion within an isostructural subspace corresponds to constant semantics, while jumps between isostructural subspaces correspond to semantic innovations (or losses).

In the drift behaviors explored above, the 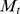 went to stasis (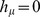) corresponding to periodic formal languages. Clearly, such a long-term condition falls far short as a model of human communication chains. The resulting communications, though distant from those at the beginning of the chain, are not periodic. To more closely capture emergent semantics in the context of sequential language learning, we have extended structural drift to include mutation and selection. In future work we will use these extensions to investigate how the former prevents permanent stasis and the latter enables a preference for intelligible phrases.

#### Cultural evolution and iterated learning

Extending these observations, the Iterated Learning Model (ILM) of language evolution [38], [39] is of particular interest. In this model, a language evolves by repeated production and acquisition by agents under cultural pressures and the “poverty of the stimulus” [38]. Via this process, language is effectively forced through a transmission bottleneck that requires the learning agent to generalize from finite data. This, in turn, exerts pressure on the language to adapt to the bias of the learner. Thus, in contrast to traditional views that the human brain evolved to learn language, ILM suggests that language also adapts to be learnable by the human brain.

ILM incorporates the sequential learning and propagation of error we discuss here and provides valuable insight into the effects of error and cultural mutations on the evolution of language for the “human niche”. There are various simulation approaches to ILM with both single and multiple agents based on, for example, neural networks and Bayesian inference, as well as experiments with human subjects. We suggest that structural drift could also serve as the basis for single-agent ILM experiments, as found in Swarup et al. [40], where populations of alleles in the former are replaced by linguistic features of the latter. The benefits are compelling: an information-theoretic framework for quantifying the trade-off between learner bias and transmission bottleneck pressures, visualization of cultural evolution via the CE diagram, and decomposition of the time-to-stasis of linguistic features in terms of isostructural subspaces as presented above.

#### Epochal evolution

Beyond applications to knowledge transmission via serial communication channels, structural drift gives an alternative view of drift processes in population genetics. In light of new kinds of evolutionary behavior, it reframes the original questions about underlying mechanisms and extends their scope to phenomena that exhibit memory in the sampling process or that derive from structure in populations. Examples of the latter include niche construction [41], the effects of environmental toxins [42], changes in predation [43], and socio-political factors [44] where memory lies in the spatial distribution of populations. In addition to these, several applications to areas beyond population genetics proper suggest themselves.

An intriguing parallel exists between structural drift and the longstanding question about the origins of *punctuated equilibrium* [45] when modeled as the dynamics of *epochal evolution* [46], [47]. The possibility of evolution's intermittent progress—long periods of stasis punctuated by rapid change—dates back to Fisher's demonstration of metastability in drift processes with multiple alleles [13].

Epochal evolution, though, presented an alternative to the view of metastability posed by Fisher's model and Wright's adaptive landscapes [48]. Within epochal evolutionary theory, equivalence classes of genotype fitness, called *subbasins*, are connected by fitness-changing *portals* to other subbasins. A genotype is free to diffuse within its subbasin via selectively neutral mutations, until an advantageous mutation drives genotypes through a portal to a higher-fitness subbasin. An increasing number of genotypes derive from this founder and diffuse in the new subbasin until another portal to higher fitness is discovered. Thus, the structure of the subbasin-portal architecture dictates the punctuated dynamics of evolution.

Given an adaptive system which learns structure by sampling its past organization, structural drift theory implies that its evolutionary dynamics are inevitably described by punctuated equilibria. Diffusion in an isostructural subspace corresponds to a period of structured equilibrium in a subbasin and subspace jumps correspond to rapid innovation or loss of organization during the transit of a portal. In this way, structural drift establishes a connection between evolutionary innovation and structural change, identifying the conditions for creation or loss of organization. Extending structural drift to include mutation and selection will provide a theoretical framework for epochal evolution using any number of structural constraints in a population.

#### Evolution of graph-structured populations

We focused primarily on the drift of sequentially ordered populations in which the generator (an 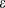-machine ) captured the structure and randomness in that ordering. We aimed to show that a population's organization plays a crucial role in its dynamics. This was, however, only one example of the general class of drift process we have in mind. For example, computational mechanics also describes structure in spatially extended systems [49], [50]. Given this, it is straightforward to build a model of drift in geographically distributed populations that exhibit spatiotemporal structure.

Though they have not tracked the structural complexity embedded in populations as we have done here, a number of investigations consider various classes of structured populations. For example, the evolutionary dynamics of structured populations have been studied using undirected graphs to represent correlations between individuals. Edge weights 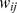 between individuals 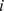 and 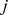 give the probability that 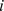 will replace 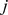 with its offspring when selected to reproduce.

By studying fixation and selection behavior on different types of graphs, Lieberman et al. found that graph structures can sometimes amplify or suppress the effects of selection, even guaranteeing the fixation of advantageous mutations [51]. Jain and Krishna [52] investigated the evolution of directed graphs and the emergence of self-reinforcing autocatalytic networks of interaction. They identified the attractors in these networks and demonstrated a diverse range of behaviors from the creation of structural complexity to its collapse and permanent loss.

Graph evolution is a model of population structure complementary to that presented by structural drift. In the latter, 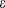-machine structure evolves over time with nodes representing equivalence classes of the distribution of selectively neutral alleles. Unlike 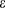-machines, the multinomial sampling of individuals in graph evolution is a memoryless process. A combined approach will allow one to examine how amplification and suppression of selection and the emergence of autocatalysis are affected by external influences on the population structure. For example, this could include how a population uses temporal memory to maintain desirable properties in anticipation of structural shifts in the environment. The result would provide a theory for niche construction in which a nonlinear dynamics of pattern formation spontaneously changes population structure.

### Final Remarks

The Fisher-Wright model of genetic drift can be viewed as a random walk of coin biases, a stochastic process that describes generational change in allele frequencies based on a strong statistical assumption: the sampling process is memoryless. Here, we developed a generalized structural drift model that adds memory to the process and examined the consequences of such population sampling memory.

Memoryful sampling is a substantial departure from modeling evolutionary processes with unordered populations. Rather than view structural drift as a replacement for the well understood theory of genetic drift, and given that the latter is a special case of structurally drifting populations, we propose that it be seen as a new avenue for theoretical invention. Given its additional ties to language and cultural evolution, we believe it will provide a novel perspective on evolution in nonbiological domains, as well.

The representation selected for the population sampling mechanism was the class of probabilistic finite-state hidden Markov models called 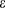-machines. We discussed how a sequential chain of 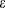-machines inferred and re-inferred from the finite data they generate parallels the drift of alleles in a finite population, using otherwise the same assumptions made by the Fisher-Wright model. The mathematical foundations developed for the latter and its related models provide a good deal quantitative, predictive power. Much of this has yet to be exploited. In concert with this, 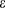-machine minimality allowed us to monitor information processing, information storage, and causal architecture during the drift process. We introduced the information-theoretic notion of structural stasis to combine the concepts of deletion, fixation, and periodicity for drift processes. Generally, structural stasis occurs when the population's allelic entropy vanishes—a quantity one can calculate in closed form due to the 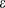-machine representation of the sampling process.

We revisited Kimura and Ohta's early results measuring the time to fixation of drifting alleles and showed that the generalized structural drift process reproduces these well known results when staying within the memoryless sampling process subspace. Starting with structured populations outside of that subspace led the sampling process to exhibit memory effects including structural innovation and loss, complex transients, and greatly reduced stasis times.

Simulations demonstrated how an 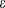-machine diffuses through isostructural process subspaces during sequential learning. The result was a very complex time-to-stasis dependence on the initial probability parameter—much more complicated than Kimura's theory describes. Nonetheless, we showed that a process' time to stasis can be decomposed into sums over these independent subspaces. Moreover, the time spent in an isostructural subspace depends on the value of the 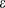-machine probability parameters at the time of entry. This suggests an extension to Kimura's theory for predicting the time to stasis for each isostructural component independently. Much of the phenomenological analysis was facilitated by the global view of drift process space given by the complexity-entropy diagram.

Drift processes with memory generally describe the evolution of structured populations without mutation or selection. Nonetheless, we showed that structure leads to substantially shorter stasis times. This was seen in drifts starting with the Biased Coin and Golden Mean Processes, where the Golden Mean jumps into the Biased Coin subspace close to an absorbing state. This suggests that even without selection, population structure and sampling memory matter in evolutionary dynamics. The temporal or spatial memory captured by the 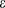-machine can be interpreted as nonrandom mating, reducing the effective population size 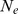 and, in doing so, increasing sampling variance. It also suggests that memoryless models restrict sequential learning and overestimate stasis times for structured populations.

We demonstrated how structural drift—diffusion, structural innovation and loss—are controlled by the architecture of connected isostructural subspaces. Many questions remain about these subspaces. What is the degree of subspace-jump irreversibility? Can we predict the likelihood of these jumps? What does the phase portrait of a drift process look like? Thus, to better understand structural drift, we need to analyze the high-level organization of generalized drift process space.

Fortunately, 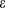-machines are in one-to-one correspondence with structured processes [25]. Thus, the preceding question reduces to understanding the space of 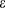-machines and how they can be connected by diffusion processes. Is the diffusion within each process subspace predicted by Kimura's theory or some simple variant? We have given preliminary evidence that it does. And so, there are reasons to be optimistic that in face of the open-ended complexity of structural drift, a good deal can be predicted analytically. And this, in turn, will lead to quantitative applications.
